# Odontogenic Fibromyxoma of the Maxilla: A Case Report and Review of the Literature

**DOI:** 10.1155/2011/238712

**Published:** 2011-05-02

**Authors:** Eva-Maria Dietrich, Styliani Papaemmanouil, Giorgos Koloutsos, Hlias Antoniades, Konstantinos Antoniades

**Affiliations:** ^1^Oral and Maxillofacial Surgery Department, General Hospital “G. Papanikolaou”, Thessaloniki, 57010 Eksoxi, Greece; ^2^Department of Pathology, General Hospital “G. Papanikolaou”, Thessaloniki, 57010 Eksoxi, Greece

## Abstract

Fibromyxoma represents a rare benign neoplasm that mostly affects the posterior region of the mandible. Here, we report the case of a 46-year-old male with a swelling of the right maxilla. After proper diagnosis, he was treated with enucleation and curettage of the tumor. The defect was filled with a pedicled buccal fat pad flap. The mesenchymal origin from the dental follicle of the fibromyxoma is the most plausible explanation. Radiological examination with MRI, CT, and conventional radiography contributes to the differential diagnosis from other benign tumors, such as the ameloblastoma. Its management is surgical and comprises enucleation and curettage or en bloc resection. Patients must be monitored for at least two years postoperatively in order to diagnose possible recurrence. According to the literature, the maxilla is a rare location of a fibromyxoma and, to our knowledge, our case is the 30th presented case of a fibromyxoma of the maxilla.

## 1. Introduction

Odontogenic fibromyxoma represents a rare slow-growing benign neoplasm, usually occurring in the 2nd and 3rd decades of life, rarely in children or adults over 50 years of age [1, 2]. It is described as a myxoma with abundant collagen fibres. Myxomas in general represent from 2.3% to 17.7% of all odontogenic tumors with fibromyxomas representing a small number of all myxomas [3]. Their size varies and in case of multilocular myxomas it may reach 4 cm [4]. They do not metastasize to the lymphatics [5]. Main sign is the swelling of the affected region and the displacement of dentition, with pain occurring less frequently mostly in cases of soft tissue myxomas [6]. Paresthesia, hypesthesia, anesthesia, or negative results of the vital tests during clinical examination are very rare [4, 7]. Although the origin of a myxoma is still obscure, an origination from the dental follicle seems to be the most reasonable explanation [1]. 

The aim of this case report and review of the literature is to present the rarity of a fibromyxoma of the maxilla, the contribution of the radiological examination to the differential diagnosis, and the importance of a meticulous enucleation in order to prevent recurrence.

## 2. Case Presentation

A 46-year-old male was referred to the outpatient department of the Oral and Maxillofacial Surgery Clinic of the General Hospital “G. Papanikolaou” of Thessaloniki, with a swelling of the right maxilla. The swelling occurred 8 months prior to the consultation. Facial and mucosal numbness, pain, or tooth mobility was absent.

**Figure 1:**
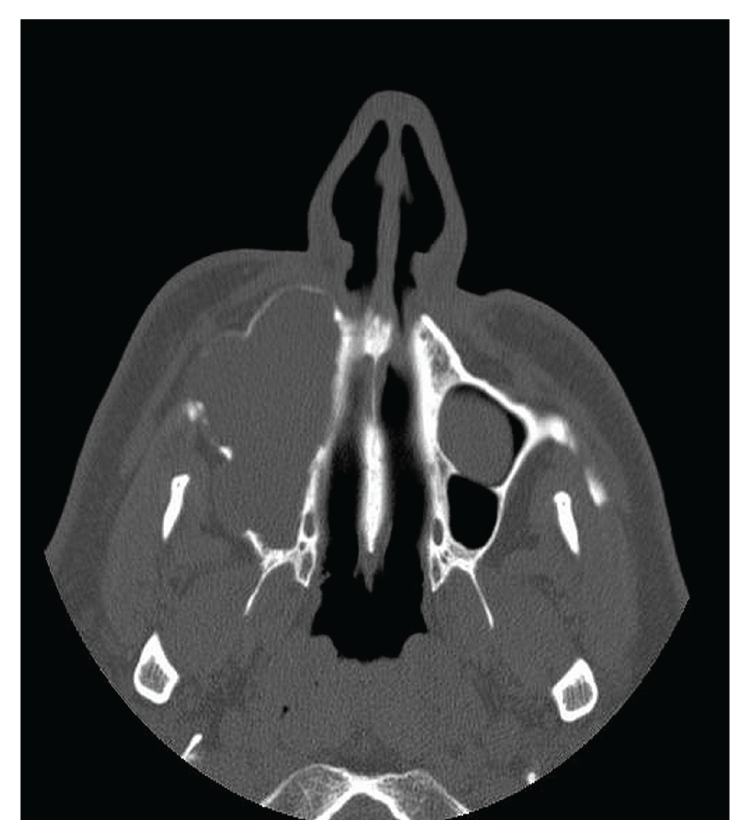
Axial Computed Tomography (CT) reveals expansion of the walls of the right maxillary sinus, obstruction with low density tissue of the whole cavity, and local erosion of the walls.

The medical anamnesis of the patient did not reveal anything in relation to the pathological condition. Radiological investigation by means of a panoramic radiograph was not helpful in diagnosing the lesion. Waters' view revealed complete obstruction of the right maxillary sinus. A Computed Tomography (CT) imaging of the maxilla revealed a large radiolucent lesion extending from the area of the right canine to the area mesial to the first molar. Examination with Computed Tomography (CT) Scan showed expansion of the walls of the right maxillary sinus, obstruction with low density tissue of the whole cavity, and local erosion of the walls (Figure 1). The intravenous administration of contrast agent showed no enhancement of the lesion. Involvement of the floor of the left maxillary sinus, partial obstruction of the ethmoid sinus, and slight thickening of the mucosa of the left frontal sinus are indicative of a secondary sinusitis. The nasopharynx and lateral pharyngeal spaces were normal.

The lesion was approached by means of a lateral rhinotomy incision, with enucleation and curettage of the tumor. 

**Figure 2:**
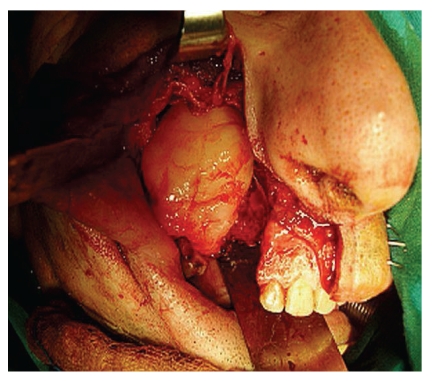
Intraoperative view.

**Figure 3:**
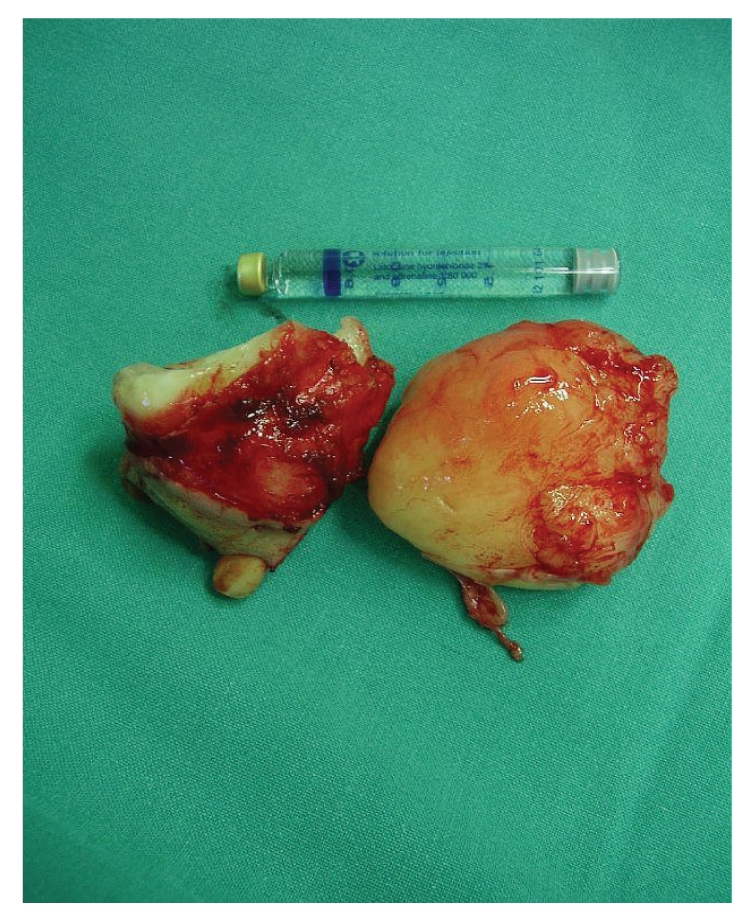
The lesion with a size of 12 × 4 cm and a solid composition.

The lesion had a solid consistency and was totally resected (Figures 2 and 3). The defect was filled with a pedicled buccal fat pad flap. 

**Figure 4:**
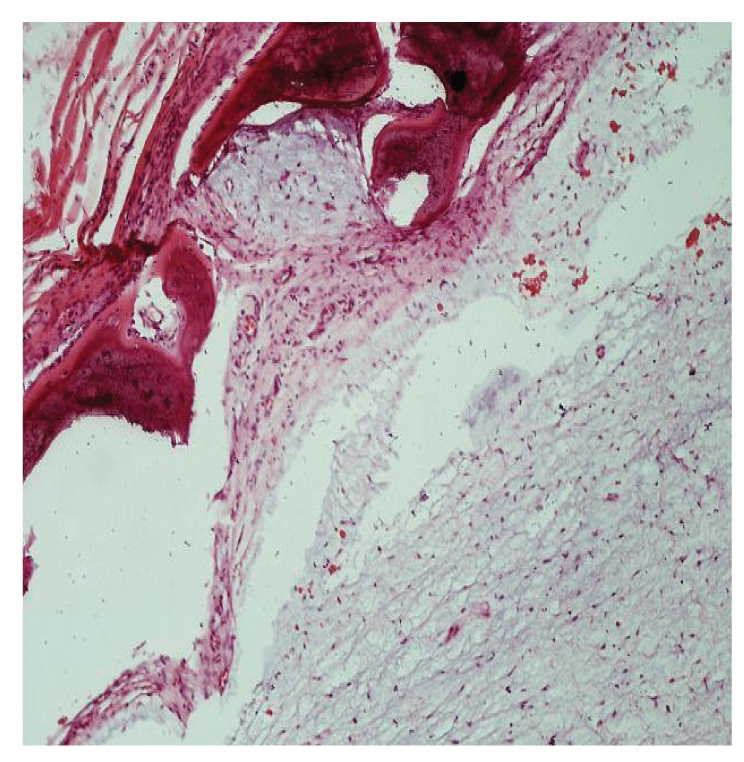
Histopathological examination revealed that randomly stellate, oval or spindle-shaped cells in a myxoid stroma, septa of residual lamellar bone and odontogenic myxoma are present into the marrow space in a pseudomalignant pattern. Variable amount of collagen fibres can be seen (x200, H + E).

The histopathological examination revealed randomly stellate, oval, and spindle-shaped cells in a myxoid stroma (Figure 4). Septa of residual lamellar bone and odontogenic myxoma were present into the marrow space in a pseudo-malignant pattern (Figure 4). Immunohistochemical examination by means of Ki-67 labeling index revealed a low rate of cell mitosis.

Two years postoperatively, the patient shows no signs of recurrence. His rehabilitation period was uneventful and he gained complete function soon after surgery.

In order to prove evidence of the rarity of a fibromyxoma of the maxilla and the frequency of recurrence, a literature search was carried out using Pubmed. Search terms included ≪fibromyxoma≫ and ≪myxoma≫. Exclusion criteria were not relevant papers, interviews, books' and conferences' abstracts, comments, replies to author and to editor, and unsupported opinion of an expert. 43 articles met our criteria. In order to record only reports of fibromyxoma and not myxoma, the articles were further sorted, in order to include those reports of fibromyxomas that were mentioned under the general term myxoma. Finally, 19 articles met all criteria and were chosen for further evaluation (Table 1) [8–26].

## 3. Discussion

Myxoma/fibromyxoma is a rare odontogenic neoplasm. Fibromyxoma is classified as a specific type of myxoma with a higher fibrous/myxoid tissue ratio than myxoma. There is a discrepancy regarding the reports of fibromyxoma, as many of them are classified under the general term “myxoma”, making the review of the literature difficult. According to Dutz and Stout, the term myxoma was first used by Virchow in 1863, but the term fibromyxoma was described by Marcove et al. in 1964 who reported extragnathic locations of fibromyxoma [27, 28]. We use the term myxoma/fibromyxoma as it is being used in many histopathological books in order to describe myxomas of the jaw bones. The review of the literature for previous reports of fibromyxoma was based on case reports that clearly report a “fibromyxoma”. 

Myxomas/fibromyxomas are usually located intraorally most often in the posterior regions of the mandible, its angle and ramus and rarely extraorally [6, 29]. The maxilla and anterior region of the mandible are rarely affected. The lesion can be diffused or well defined, uni- or multilocular. It is characterized by a mucous or gelatinous grayish-white tissue that replaces the spongy bone and displaces the cortical plates of the jaws [1]. Root displacement and resorption may be present [1]. It may refer to hard and also to soft tissues. 

Previous theories stress that the lesion derives from the neural sheath or is the result of degeneration of fibromas, lipomas and so forth, due to the chronic irritation and the degenerative processes following tissue anoxemia [26]. Recent studies advocate that myxomas/fibromyxomas arise from the mesenchymatous tissue of the dental follicle, thus being described as odontogenic with fibroblasts playing the major role in cell dispersal [1]. This explanation fails to describe soft tissue myxomas [7]. They probably arise from supportive structures of the teeth like the gingiva and the periodontal ligament [7]. 

Histopathological characteristics of the myxoma/fibromyxoma are the hypocellularity, the presence of stellate, spindle-shaped cells into a loose myxoid extracellular matrix with cells presenting with thin, long cytoplasmic prolongations that give to the tissue characteristics of immature mesenchyma [30]. The fibromyxoid lesion may present loci of calcification or ossification and a higher amount of collagen fibres and vessels than a typical myxoma [14]. The presence of cells positive for actin fibres suggests that myofibroblasts may play a crucial role in cell proliferation in cooperation with the islands of odontogenic epithelium and mast cells [4, 7]. 

Myxomas are diagnosed with radiological, histological, and histochemical investigation. The radiological investigation reveals homogenous radiolucencies or sclerotic trabeculations with different appearances, like “honeycomb”, “soap bubble”, and “tennis racket” [31]. In our case, the lesion appeared as a large radiolucent area with no trabeculations. 

Radiological examination plays a crucial role for the differential diagnosis of myxomas/fibromyxomas and also between benign myxomas and malignant neoplasms with myxomatous tissue. In Magnetic Resonance Imaging (MRI), the lesion shows low-signal intensity in T1 and high-signal intensity in T2 [5]. In contrast, Kawai et al. advocate that the high-signal is shown in T1 and not in T2 [31]. These discrepancies may be related to the ratio of fibrous/myxoid tissue, the viscosity, the concentration of proteins, the presence of haemorrhage and the hypocellularity [5, 31]. Immunohistochemical examination uses antibodies against specific biological substances of neuronal, muscular, epithelial, and mesenchymal tissues. The evaluation of the presence of vimentin, an intermediate filament of the cytoskeleton characterises mesenchymal tissues, thus also myxomas [1]. Fibromyxomas also contain a high amount of hyaluronic acid [32].

During the process of differential diagnosis pathological conditions that should be included are ameloblastoma, central haemangioma, fibrous dysplasia, odontogenic cysts, aneurysmal cysts, central gigantocytic granuloma, metastatic neoplasms, well-differentiated liposarcoma, and other rare entities like desmoplastic fibroma [5, 33].

The main pathological condition that may lead to difficulties in diagnosis is the ameloblastoma, especially when the bony septa are curviform [3]. An important characteristic for differential diagnosis is the fact that when a contrast agent (Gd-DTPA) is being administered, in case of the ameloblastoma the MRI shows strong enhancement of the solid portion of the tumor, in contrast to the myxoma that shows homogenous high signal intensity [3]. It is also important to mention that root displacement and resorption is not unique in ameloblastoma.

The treatment of the fibromyxoma is surgical and involves enucleation and curettage. The avoidance of recurrence is strongly related to the complete resection of the lesion. The patient should be monitored for at least two years after the surgical intervention due to the higher rate of recurrence during this period [5]. 

Myxomas/fibromyxomas show a recurrence rate between 25% [2] and 43% [1]. This is strongly related to the nature of the lesion, presenting without a sheath, thus making the complete removal difficult. Other odontogenic tumors, like the keratocyst or the ameloblastoma show a higher recurrence rate of 30% [34]–58,3% [35] and 55%–90%, respectively [35]. The frequency of recurrence of a fibromyxoma of the jaws is higher than that of any other bone thus having a poorer prognosis [36].

It is stressed that complete resection and peripheral osteotomy is the treatment of choice depending on the size and behaviour of the tumor and results in a lower rate of recurrence [6, 7, 33, 37]. Simon et al. suggest that radical resection with a margin of 1,5–2 cm of healthy bone is the treatment of choice [6]. Small bony defects of the maxilla, under 5 cm, can be reconstructed by means of a pedicled buccal fat pad flap (BFP) [38, 39]. Greater bony defects require the positioning of an obturator prior to the reconstruction with a graft.

In conclusion, the maxilla is a rare location of a fibromyxoma. The radiological examination by means of CT and MRI plays an important role in the diagnosis of a fibromyxoma and in the differential diagnosis from other pathological entities such as the ameloblastoma. Its management is surgical and ranges from enucleation and curettage to complete resection and peripheral osteotomy according to its size. Patients must be monitored for at least two years postoperatively in order to diagnose possible recurrence.

## Figures and Tables

**Table 1:** Reported cases of fibromyxoma of the maxilla.

Case report	Number of patients	Radiographic appearance
Infante-Cossío et al., 2010 [8]	1	Multilocular expansile radiolucent lesion of right maxilla, that destroys the buccal and palatal cortical bone

Singaraju et al., 2010 [9]	1	Unilocular expansile radiolucent lesion of right maxilla and antrum with teeth displacement and root resorption

Veras Filho et al., 2008 [10]	1	Multilocular bone destruction of ill-defined margins and involvement of the left maxillary sinus

Sivakumar et al., 2008 [11]	1	Multilocular expansile radiolucent lesion of the right maxilla with “tennis racket” appearance that involves the antrum

Berry and Puri, 2006 [12]	1	Lesion that destroys the right maxilla completely and extends into the right infratemporal fossa

Mishra et al., 2004 [13]	1	Expansion of right alveolar margin without bony erosion and involvement of the maxillary sinus

Keszler et al., 1995 [14]	3	Unilocular lesion with cortical expansion and tooth displacement, tennis racket-like or soap bubble image

Abiose et al., 1987 [15]	4	Multilocular or honeycombed lesion with varying degrees of root resorption

Schneider and Weisinger, 1985 [16]	1	Radiolucent area of the right maxilla within the periodontal ligament with alveolar bone resorption and tooth displacement

Kabir et al., 1985 [17]	1	Destruction of the medial wall of the right maxillary antrum and right upper alveolus

Prasad and Sharan, 1983 [18]	1	Erosion of the right anterolateral wall of the maxilla, obstruction of the maxillary antrum

Russell et al., 1979 [19]	1	Mixed radiopacity and radiolucency and divergence of roots

Cho et al., 1973 [20]	6	Multilocular or honeycombed lesions

Harrison and Eggleston, 1973 [21]	1	Opacification of the right maxillary antrum, destruction of the lateral wall, and new bone formation on the lateral aspect of the right maxillary alveolus

Kakar and Sood, 1969 [22]	1	Honeycomb appearance

Buchner and Ramon, 1965 [23]	1	Multilocular radiolucent area of left maxilla, that extends from the midline to the region of the molars

Archer, 1960 [24]	1	Irregular radiopaque and radiolucent patterns of left maxilla, anterior to an unerupted impacted third molar

Bruce and Royer, 1952 [25]	1	Radiolucent area with fine angular trabeculations of left maxilla

Wawro and Reed, 1950 [26]	1	Large soft tissue mass that destroys the alveolar process, the zygoma, the floor of the orbit, and the right ethmoid cells
